# Supplementary material for: Determination of human DNA replication origin position and efficiency reveals principles of initiation zone organisation
Source: Nucleic Acids Res. 2022 Jul 8;50(13):7436–50. doi: 10.1093/nar/gkac555 (PMC9303276; doi:10.1093/nar/gkac555)
Supplement: gkac555_Supplemental_File [file gkac555_supplemental_file.docx]

**SUPPLEMENTARY INFORMATION FOR:**

**Determination of human DNA replication origin position and efficiency reveals principles of initiation zone organisation**

Guillaume Guilbaud^1*^, Pierre Murat^1^, Helen S. Wilkes^2^, Leticia Koch Lerner^1,3^, Julian E. Sale^1*^ & Torsten Krude^2*^

^1^ Division of Protein & Nucleic Acid Chemistry, MRC Laboratory of Molecular Biology, Francis Crick Avenue, Cambridge, CB2 0QH, U.K.

^2^ Department of Zoology, University of Cambridge, Downing Street, Cambridge, CB2 3EJ, U.K.

^3^ Present address: Centre de Recherche des Cordeliers, 15 rue de l’Ecole de Médecine 75006 Paris, France

^*^ to whom correspondence may be addressed: [guilbaud@mrc-lmb.cam.ac.uk](mailto:guilbaud@mrc-lmb.cam.ac.uk); [jes@mrc-lmb.cam.ac.uk](mailto:jes@mrc-lmb.cam.ac.uk); [tk218@cam.ac.uk](mailto:tk218@cam.ac.uk)

**Figure S1.**

**
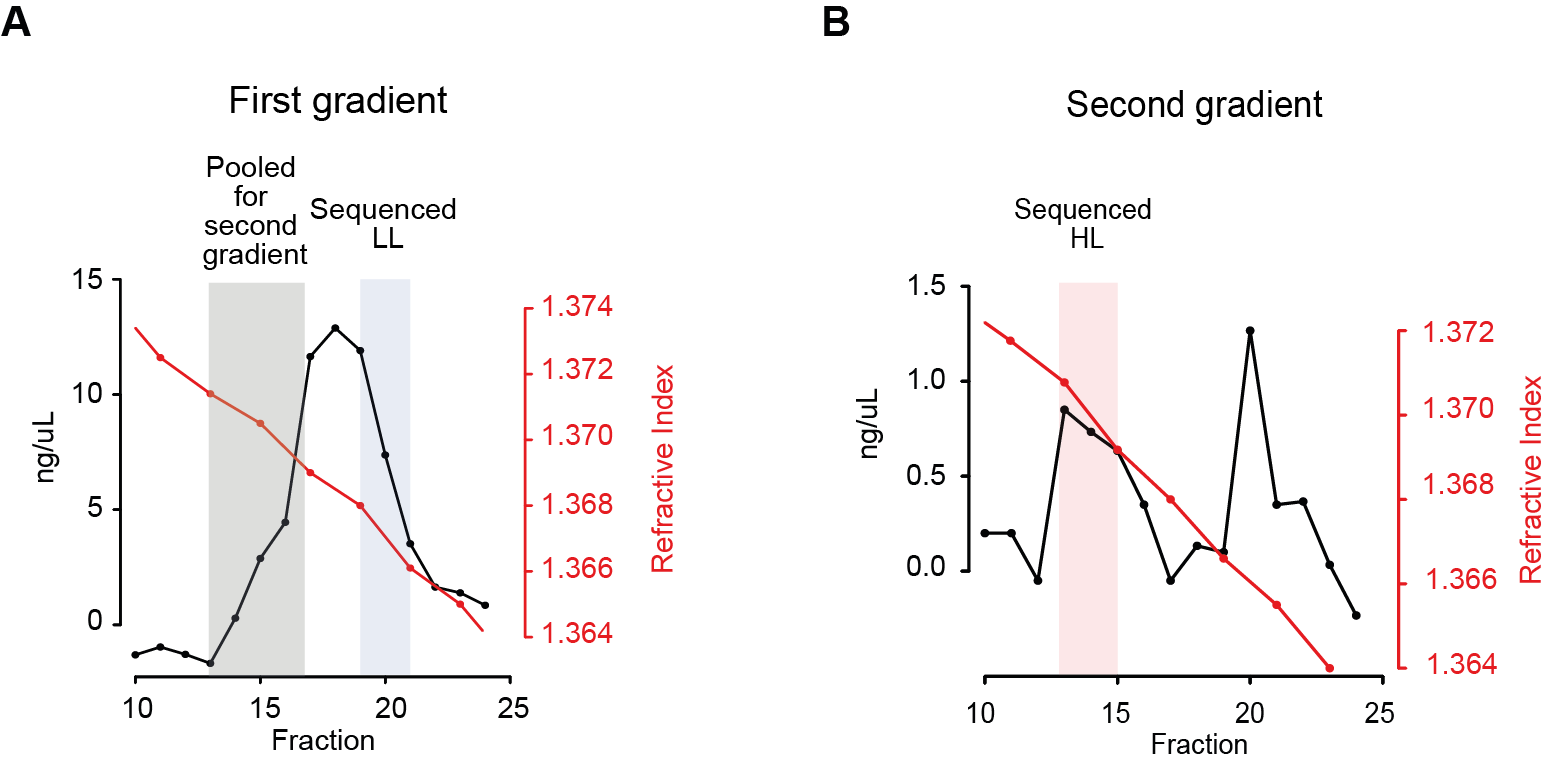
**

**Separation of HL and LL DNA fractions by two sequential caesium sulphate density gradients.** Examples of representative density gradients with DNA from a 3 hour incubation are shown. A. First density gradient to fractionate bulk DNA after density substitution and fragmentation. Fractions of LL DNA used for sequencing (refractive index RI = 1.3660 - 1.3680; density D = 1.422 – 1.446) are indicated by blue shading. Fractions containing HL DNA pooled for further separation on a second gradient (RI = 1.3695 – 1.3714; D = 1.464 – 1.487) are shaded grey. B. Second density gradient to remove contaminating LL DNA from the pooled HL DNA of the first gradient. Peak fractions of HL DNA used for sequencing (RI = 1.3693 - 1.3713; D = 1.461 – 1.485) are shaded red. Black: measured DNA content of each fraction; Red: measured RI values of each other fraction.

For reference, in caesium sulphate gradients the densities of non-substituted genomic DNA of different species have been reported as D = 1.419 – 1.435, for GC contents of 25 – 71% GC (D= 1.422 for human DNA, 41% GC) (1). The pooled LL fractions used for sequencing cover this range. Replicated double stranded M13 DNA (41% GC) that is hemisubstituted with BrdUTP (HL) has a peak RI of 1.3700 (corresponding to D = 1.470) (2, 3). The pooled HL fractions used for sequencing are centered on this value.

**Figure S2.**


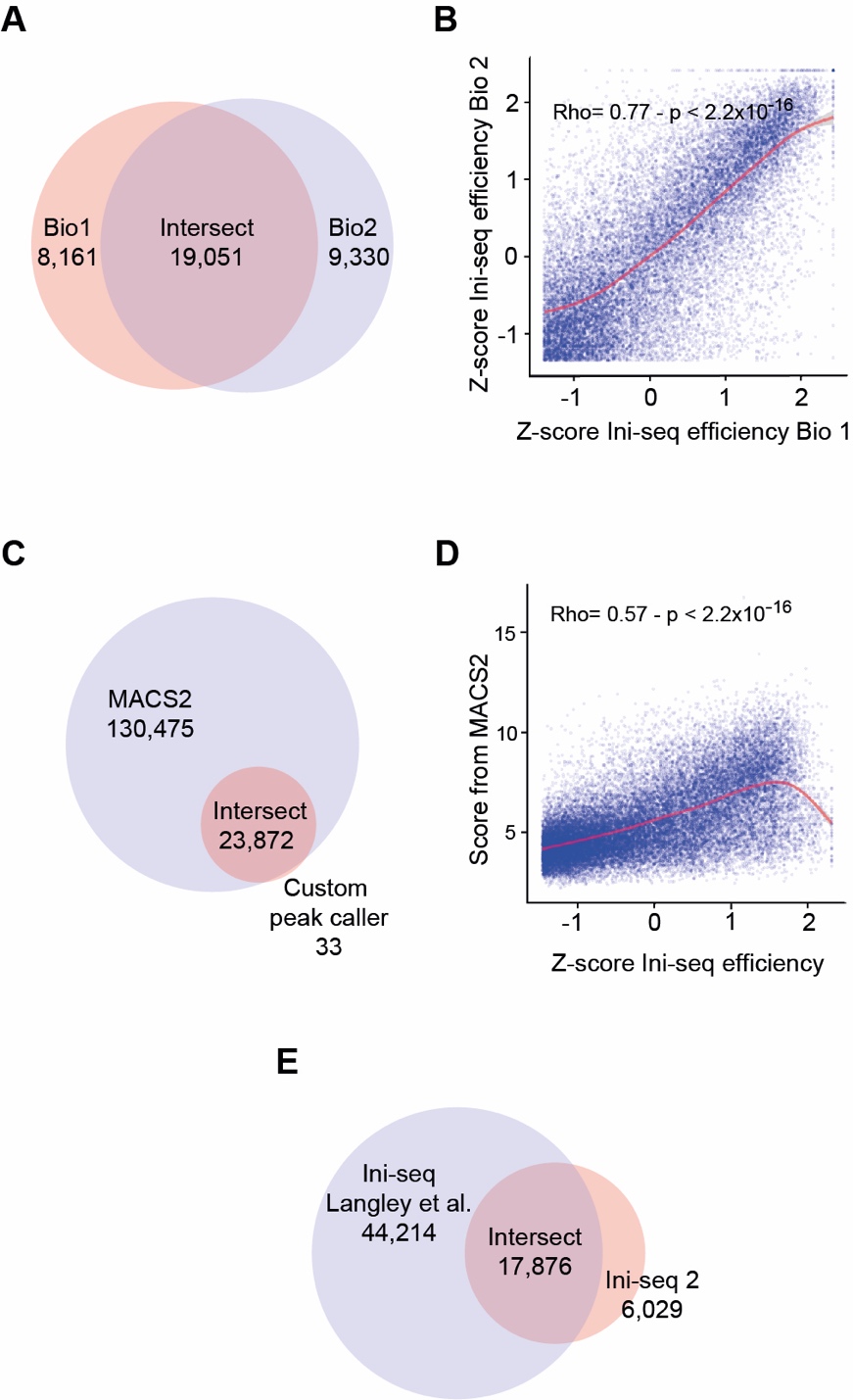


**Biological replicates of ini-seq 2 and comparison between peak callers.** A. Venn diagram of origins called in the two biological replicate ini-seq 2 experiments (terms Bio1 and Bio2). Permutation test p = 0.0001, Z-score 1256. B. Correlation of origin efficiency in the intersect of the biological replicates. Correlation: Pearson. C. Venn diagram of origins called by the custom peak caller described in this paper compared with MACS2 (4) using the parameters of Akerman et al. (5). Permutation test p = 0.0001, Z-score 538. D. Correlation of origin efficiency in the intersect of (C). Correlation: Pearson. E. Venn diagram of origins called in ini-seq 2 compared with the larger biological replicate of the original ini-seq experiments (6). Permutation test p = 0.0001, Z-score 286.

**Figure S3.**

**
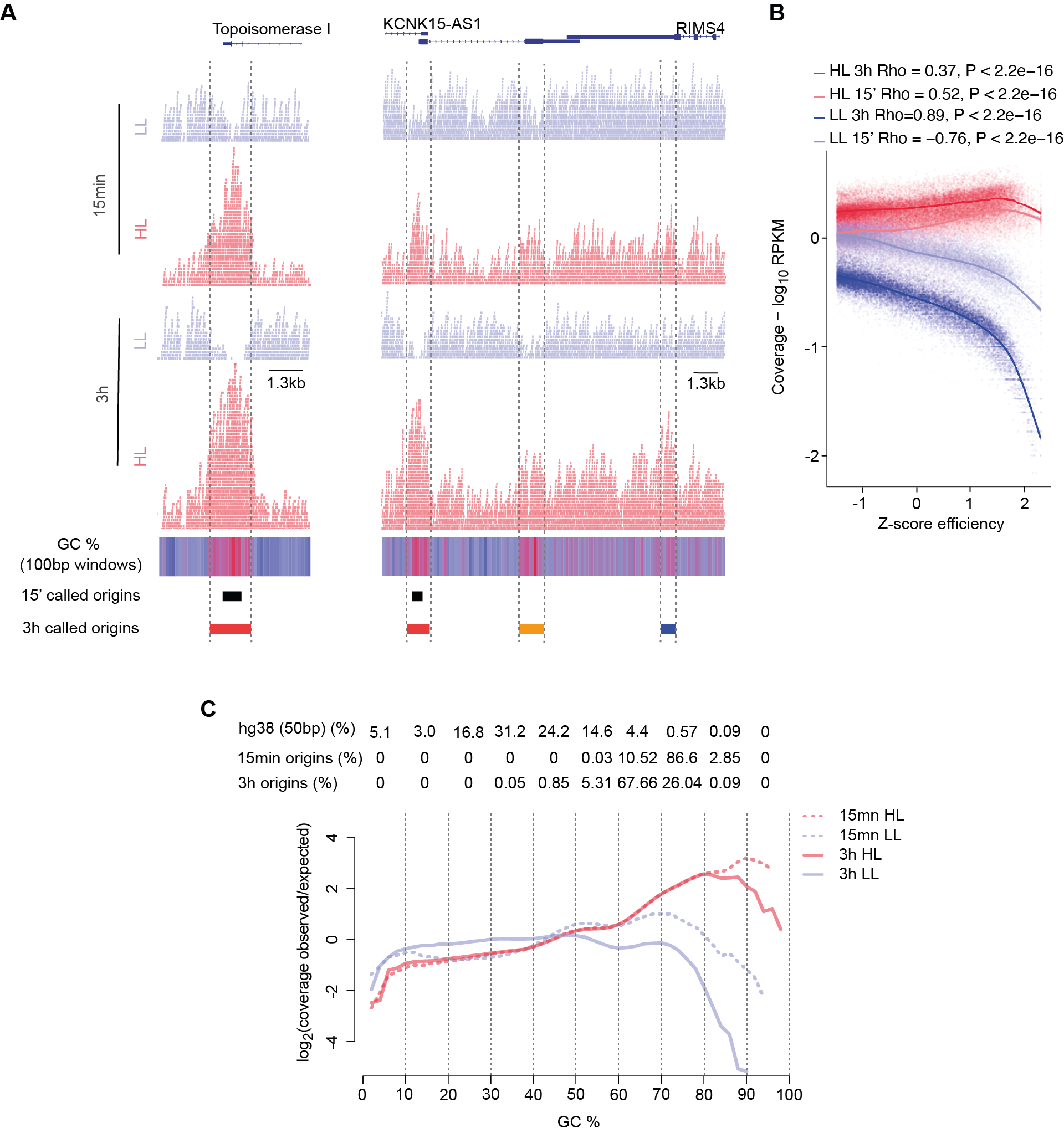
**

**Evolution of HL and LL read counts as a function of time, origin efficiency and GC content.** A. An IGV screenshot of raw mapped reads demonstrating enrichment of HL reads (red) and depletion of LL reads (blue) reads for the genomic regions on chromosome 20 shown in Figure 1. The GC content is shown as a heatmap (blue gradient < 50% GC; red gradient >50% GC). The called origins are shown at 15 minutes and 3 hours. For the 3-hour origins, high efficiency = red, medium efficiency = orange and low efficiency = blue. B. Read coverage at 15 minutes and 3 hours for each origin called at 3 hours as a function of efficiency determined at 3 hours. Red = HL, blue = LL. Correlation: Pearson. C. Genome wide read coverage at 15 minutes and 3 hours for each origin called at 3 hours as a function of GC content. Read coverage is presented as the ratio of observed over expected as computed by the deepTools command computeGCbias (7). Numbers above the panel report: Top line: the fraction of the hg38 genome (segmented of 50bp windows) falling in each decile of GC abundance expressed as a percentage; Second line: the fraction of origins detected at 15 minutes falling in each decile of GC abundance; Third line: the fraction of origins detected at 3 hours falling in each decile of GC abundance. The GC abundance was computed with the bedtools nuc function.

**Figure S4.**

**
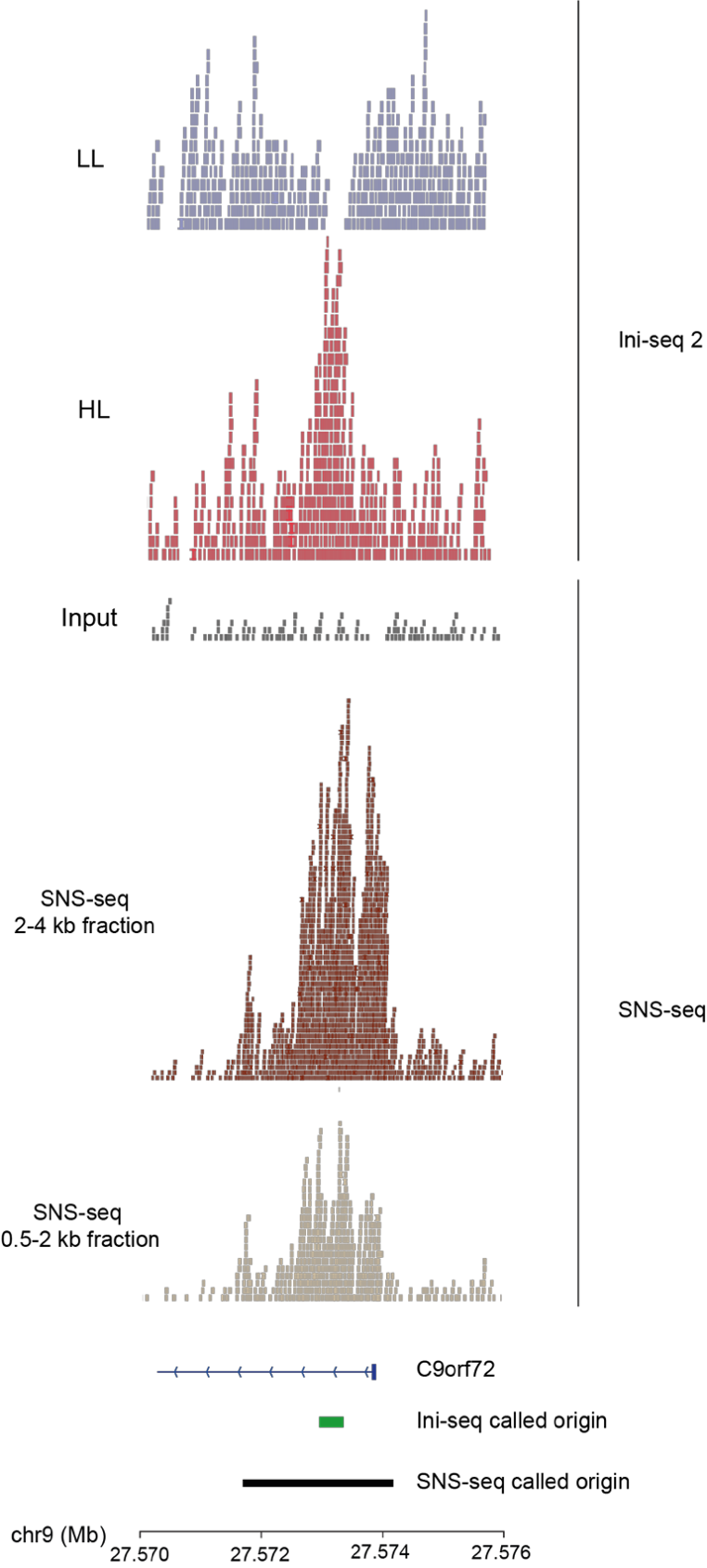
**

**Example IGV** raw mapped reads **tracks comparing ini-seq 2 with SNS-seq in EJ30 cells.** A region at the start of the C9ORF72 locus on Chr 9 is shown. From top to bottom: Ini-seq 2 LL and HL fractions, the input (total genomic DNA) used for SNS-seq, SNS-seq reads from size fractions 2 – 4kb and 0.5 – 2 kb. The ini-seq 2 peak called with our custom peak caller is shown as a green horizontal bar; the SNS-seq peak called with MACS2 is shown in black.

**Figure S5.**

**
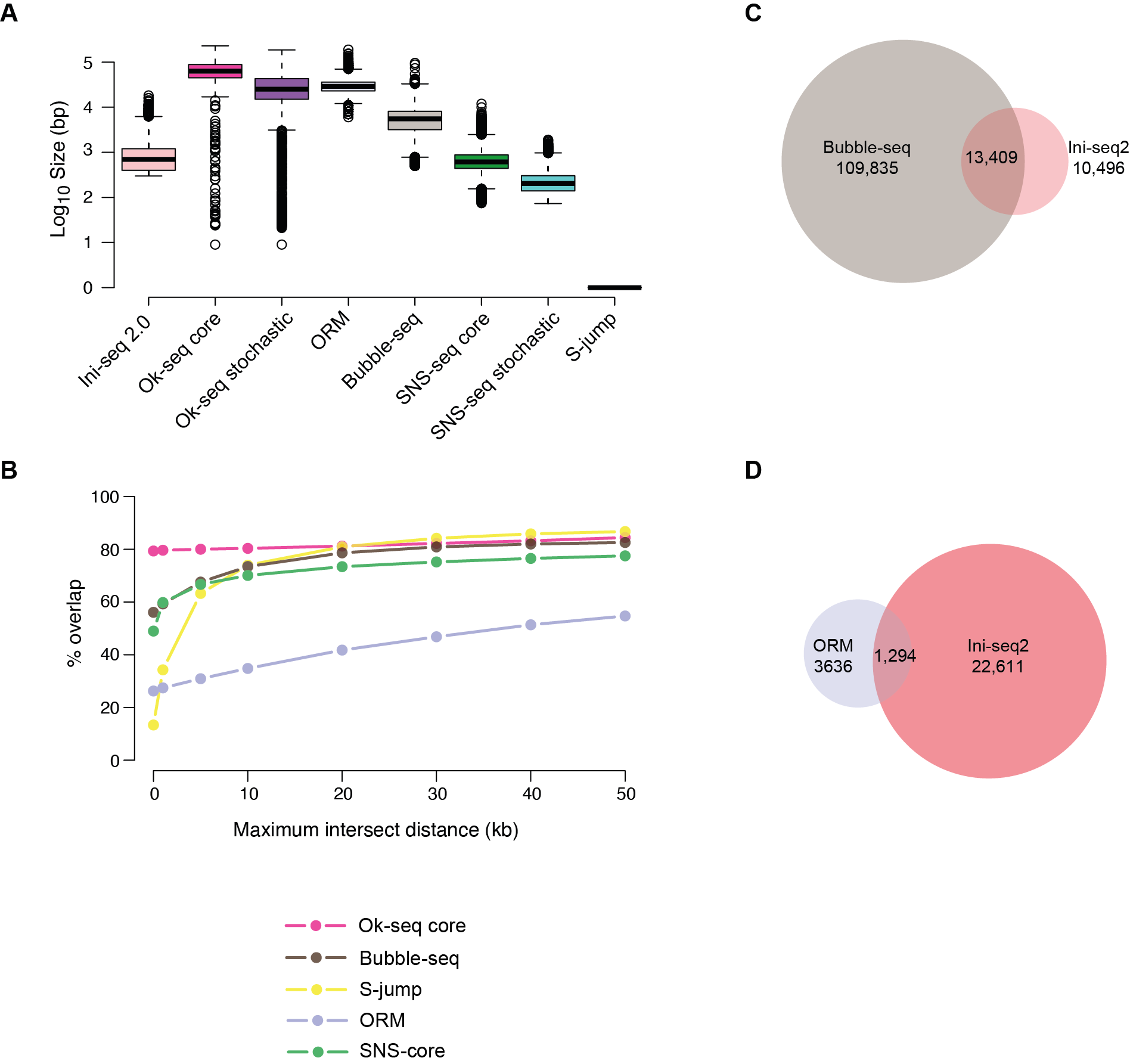
**

**Further comparison of ini-seq 2 with other origin mapping techniques.** A. Size distributions of origins called by the indicated different techniques. Whiskers represent interquartile ranges. B. Intersect between ini-seq 2 peaks and peaks called by other techniques as a function of maximum intersect distance allowed. The Y-axis gives the percentage overlap of the group with the smallest number of origins in each comparison. C. Venn diagram showing the overlap between ini-seq 2 origins and bubble-seq initiation sites (8). Maximum intersect distance allowed for overlap: 5kb. Permutation test p = 0.0001, Z-score 101. D. Venn diagram showing the overlap between ini-seq 2 origins and optical replication mapping (9). Maximum intersect distance allowed for overlap: 0kb. Permutation test p = 0.0001, Z-score 35.

**Figure S6.**

**
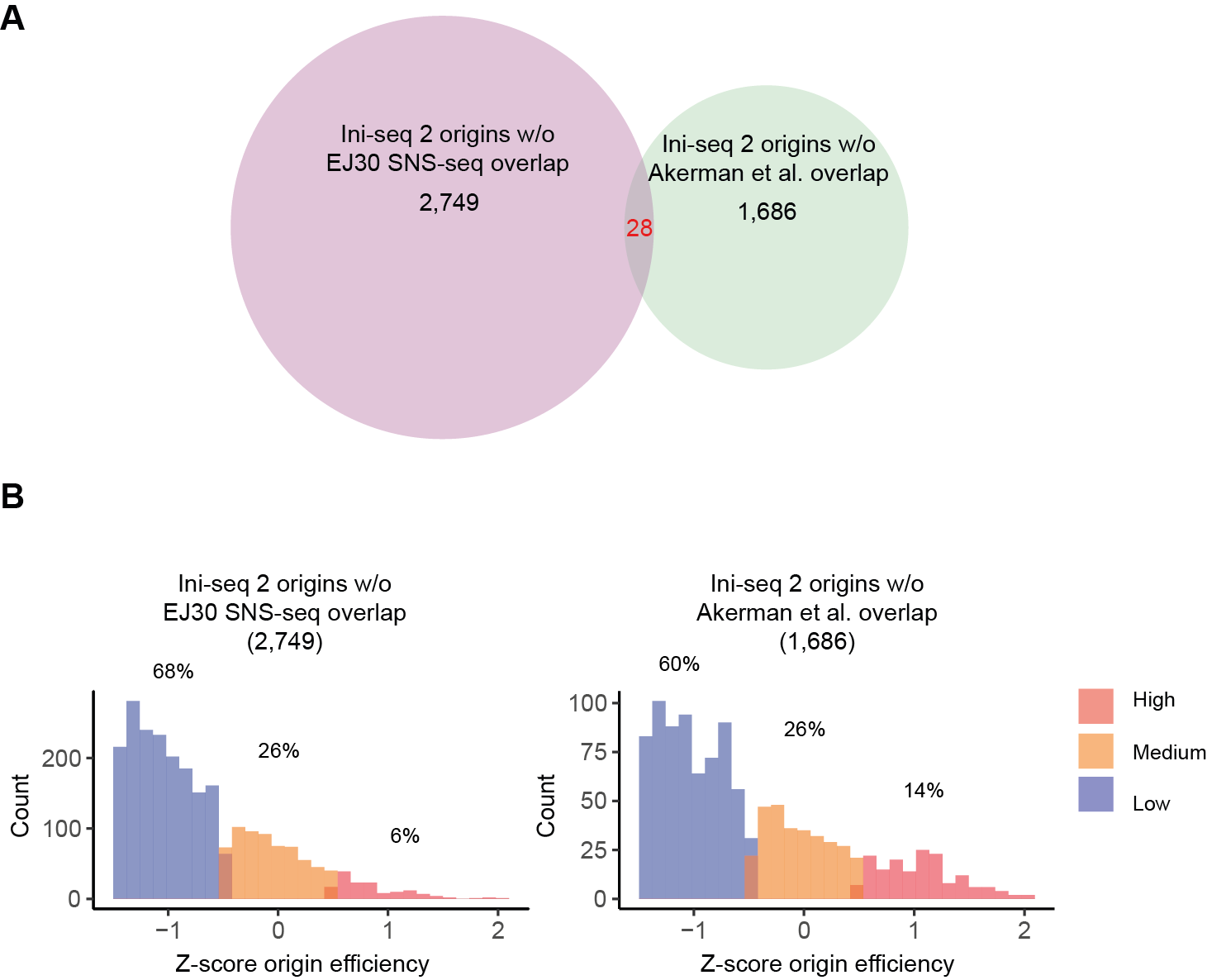
**

**Ini-seq 2 origins not found in SNS-seq experiments are enriched in the low efficiency class.** A. Venn diagram showing the overlap in the identity of ini-seq 2 origins that are not identified in our EJ30 SNS-seq experiment (purple) or in the full SNS-seq dataset of Akerman et al. (5) (green). B. The distribution of these origins by their ini-seq 2 efficiency class. Both distributions show a statistically significant bias towards the low efficiency class (Chi-square test p < 2.2 x10^-16^ taking the expected distribution as 1/3^rd^ in each group).

**Figure S7.**

**
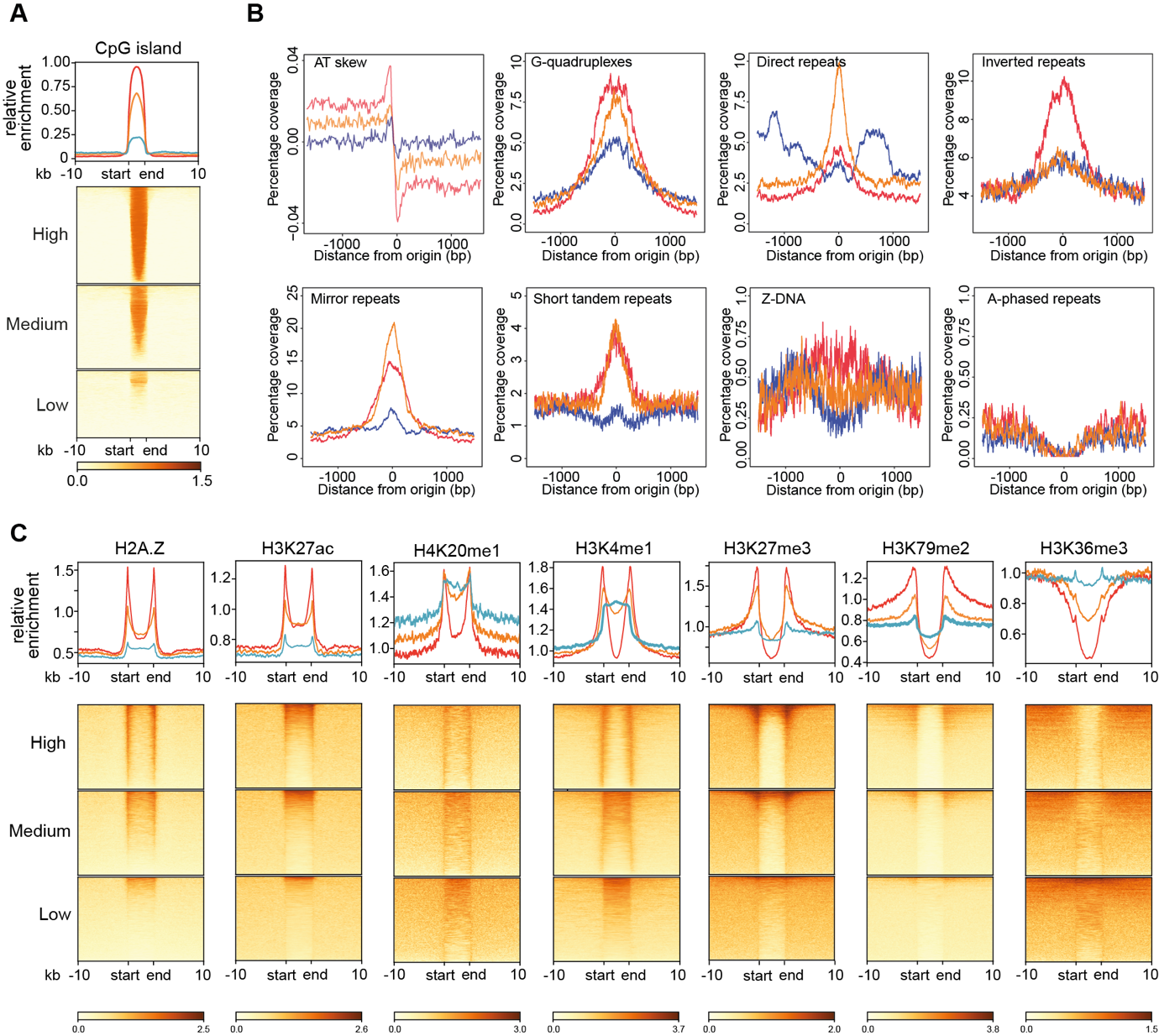
**

**Genetic and epigenetic features of ini-seq 2 replication origins.** A. Relative enrichment of CpG islands within and +/- 10kb around the ini-seq 2 origin classes of low (blue), medium (orange) and high (red) efficiency. The origin itself is represented as a metagene by ‘start’ and ‘end’. B. Distribution of AT skew, G quadruplex-forming sequences, direct repeats, inverted repeats, mirror repeats, short tandem repeats (2 – 6 bp repeat), Z-DNA and A-phased repeats (10) around origins of the low (blue), medium (orange) and high (red) efficiency classes. Areas of +/- 1200 bp of the ini-seq 2 origins are shown. C. Relative enrichment or depletion of H2A.Z, H3K27ac, H4K20me1, H3K4me1, H3K27me3, H3K79me2 and H3K36me3 within and +/- 10kb around the ini-seq 2 origin classes of low (blue), medium (orange) and high (red) efficiency. The origin itself is represented as a metagene by ‘start’ and ‘end’.

**Figure S8.**

**
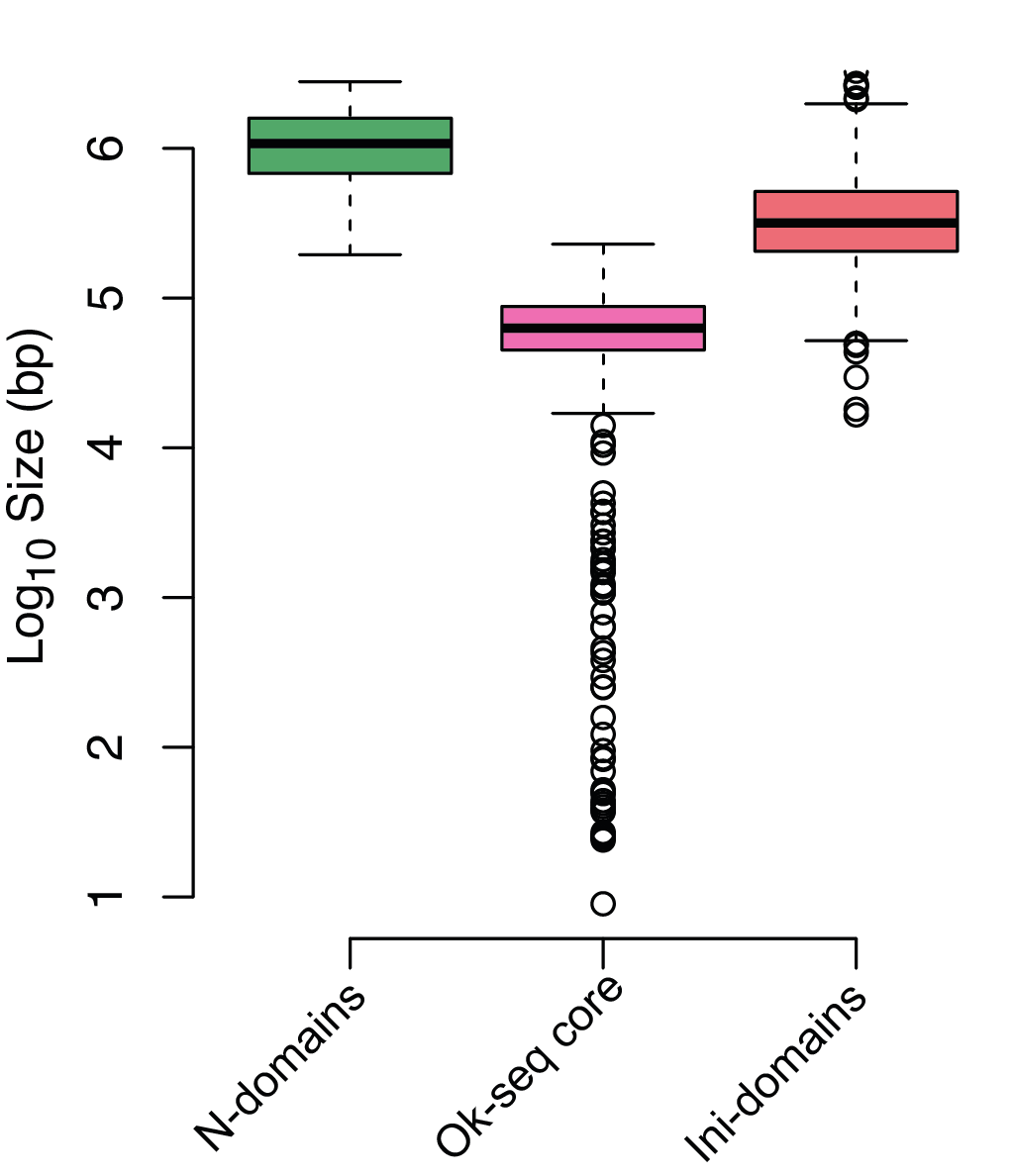
**

**Size distributions of N-domains, Ok-seq core initation zones and ini-domains.** Whiskers represent interquartile ranges.

**Supplementary References**

1. Szybalski,W. (1968) Use of cesium sulfate for equilibrium density gradient centrifugation (ed.), *Methods in enzymology 12*. Elsevier, pp. 330-360.

2. Krude,T. and Knippers,R. (1993) Nucleosome assembly during complementary DNA strand synthesis in extracts from mammalian cells. *J Biol Chem* **268**, 14432-14442.

3. Krude,T. and Knippers,R. (1994) Minichromosome replication in vitro: inhibition of re-replication by replicatively assembled nucleosomes. *J Biol Chem* **269**, 21021-21029.

4. Feng,J., Liu,T., Qin,B., Zhang,Y. and Liu,X.S. (2012) Identifying ChIP-seq enrichment using MACS. *Nat Protoc* **7**, 1728-1740.

5. Akerman,I., Kasaai,B., Bazarova,A., Sang,P.B., Peiffer,I., Artufel,M., Derelle,R., Smith,G., Rodriguez-Martinez,M., Romano,M., Kinet,S., Tino,P., Theillet,C., Taylor,N., Ballester,B. and Méchali,M. (2020) A predictable conserved DNA base composition signature defines human core DNA replication origins. *Nat Commun* **11**, 4826.

6. Langley,A.R., Gräf,S., Smith,J.C. and Krude,T. (2016) Genome-wide identification and characterisation of human DNA replication origins by initiation site sequencing (ini-seq). *Nucleic Acids Res* **44**, 10230-10247.

7. Ramírez,F., Ryan,D.P., Grüning,B., Bhardwaj,V., Kilpert,F., Richter,A.S., Heyne,S., Dündar,F. and Manke,T. (2016) deepTools2: a next generation web server for deep-sequencing data analysis. *Nucleic Acids Res* **44**, W160-5.

8. Mesner,L.D., Valsakumar,V., Cieslik,M., Pickin,R., Hamlin,J.L. and Bekiranov,S. (2013) Bubble-seq analysis of the human genome reveals distinct chromatin-mediated mechanisms for regulating early- and late-firing origins. *Genome Res* **23**, 1774-1788.

9. Wang,W., Klein,K.N., Proesmans,K., Yang,H., Marchal,C., Zhu,X., Borrman,T., Hastie,A., Weng,Z., Bechhoefer,J., Chen,C.L., Gilbert,D.M. and Rhind,N. (2021) Genome-wide mapping of human DNA replication by optical replication mapping supports a stochastic model of eukaryotic replication. *Mol Cell* S1097-2765(21)00408.

10. Haran,T.E. and Mohanty,U. (2009) The unique structure of A-tracts and intrinsic DNA bending. *Q Rev Biophys* **42**, 41-81.
